# Similarities between the effect of SARS-CoV-2 and HCV on the cellular level, and the possible role of ion channels in COVID19 progression: a review of potential targets for diagnosis and treatment

**DOI:** 10.1080/19336950.2020.1837439

**Published:** 2020-10-22

**Authors:** Hani Alothaid, Mohammed S. K. Aldughaim, Karim El Bakkouri, Sufana AlMashhadi, Ahmed A. Al-Qahtani

**Affiliations:** a Department of Basic Sciences, Faculty of Applied Medical Sciences, Al-Baha University, Al-Baha, Saudi Arabia; b Research Center, King Fahad Medical City, Riyadh, Saudi Arabia; c Rapid Test Development Department, SciMed Services and Solutions, Brussels, Belgium; d McGovern Institute for Brain Research, Massachusetts Institute of Technology, Cambridge, USA; e Department of Infection and Immunity, Research Centre, King Faisal Specialist Hospital & Research Centre, Riyadh, Saudi Arabia; f Department of Microbiology and Immunology, School of Medicine, Alfaisal University, Riyadh, Saudi Arabia

**Keywords:** COVID19, SARS-COV-2, HCV, ion channels, immune responses, peg-IFN

## Abstract

The COVID-19 pandemic, caused by severe acute respiratory syndrome coronavirus 2 (SARS-CoV-2), has prompted an urgent need to identify effective medicines for the prevention and treatment of the disease. A comparative analysis between SARS-CoV-2 and Hepatitis C Virus (HCV) can expand the available knowledge regarding the virology and potential drug targets against these viruses. Interestingly, comparing HCV with SARS-CoV-2 reveals major similarities between them, ranging from the ion channels that are utilized, to the symptoms that are exhibited by patients. Via this comparative analysis, and from what is known about HCV, the most promising treatments for COVID-19 can focus on the reduction of viral load, treatment of pulmonary system damages, and reduction of inflammation. In particular, the drugs that show most potential in this regard include ritonavir, a combination of peg-IFN, and lumacaftor-ivacaftor. This review anaylses SARS-CoV-2 from the perspective of the role of ion homeostasis and channels in viral pathomechanism. We also highlight other novel treatment approaches that can be used for both treatment and prevention of COVID-19. The relevance of this review is to offer high-quality evidence that can be used as the basis for the identification of potential solutions to the COVID-19 pandemic.

## Introduction

Coronavirus disease 2019 (COVID-19) is a highly infectious disease that is caused by severe acute respiratory syndrome coronavirus 2 (SARS-CoV-2). The disease is novel and was identified for the first time in Wuhan in December 2019 [1]. Since then, it has spread to most parts of the world, leading to the coronavirus pandemic. Though little is known about this disease, individuals affected show symptoms that include sore throat, shortness of breath, coughing, and fever. Other symptoms can include muscle pain and fatigue [2]. As a respiratory disease, the average time before an affected person develops noticeable symptoms ranges from two to fourteen days.

In other cases, symptoms such as conjunctival congestion, hemoptysis, and diarrhea may occur. As with other coronavirus infections, COVID-19 is characterized alongside respiratory infections, which include pneumonia and influenza [3]. Individuals with preexisting underlying diseases, such as lung infections, diabetes, and cardiovascular disease are likely to be more severely affected when infected with COVID-19 compared to those without preexisting underlying illnesses. Interestingly, upon infection, individuals may also show symptoms of cystic fibrosis (CF) such as sinusitis, pneumonia, and coughing. It is also possible for the patients to experience altered electrolyte levels, and this could have significant effects on sufferers and their quality of life. For instance, electrolyte imbalance can aid the progression of existing conditions such as diabetes mellitus, severe cardiovascular events, and acute or chronic renal failures [4]. This is consistent with the idea that ion transport systems, such as ion channels, are involved in the onset of COVID-19.

At the time of writing, COVID-19 is regarded as a pandemic and is ongoing. Millions of cases have been reported worldwide, and these have had significant implications on communities and economies. The number of deaths continues to increase, and the impact of the pandemic is becoming grave in many countries across the world. Notably, SARS-CoV-2 belongs to the same virus group as Middle East Respiratory Syndrome (MERS) and SARS-CoV. Nevertheless, it has certain unique characteristics that distinguish it from all other viruses. As a novel virus, little is known about SARS-CoV-2, and research is being carried out at a massive scale to understand its pathomechanism and to develop strategies that could help manage it successfully. It is thus critical to compare the impact of this condition with that of others and devise approaches that could help to better understand the disease. For example, the hepatitis C virus (HCV) is a well-studied virus with therapeutics having been developed for its treatment. It is also structurally similar to SARS-CoV-2 (discussed below). Thus comparing SARS-CoV-2 with the hepatitis C virus could allow a greater understanding of the pathophysiology of the novel coronavirus and the available options to counter it. As noted in the abstract, this is the main aim of the review, along with an exploration of the possible roles of ion channels and channelopathies as a therapeutic target in other viral diseases, which may also be extended to SARS-CoV-2.

## Discussion

### Novel coronavirus

The term novel coronavirus is an impermanent name that is usually given to coronaviruses with medical importance before characterization of the viruses that result in eventual naming. In most cases, coronaviruses are endemic in humans, as with the common cold. However, when cross-species infections occur, they produce highly virulent strains that can have huge medical implications on the community. Such strains often cause viral pneumonia, which may, in some cases, progress to acute respiratory distress syndrome and even death [5].

The examination of the viral structure of SARS-CoV-2 has been revealed to contain four structural proteins. These include the nucleocapsid, spike, membrane, and envelope proteins. The glycoprotein of the spike has antigenic properties and facilitates antigenic interaction with the host’s cell surface receptors [6]. The virus is a single-stranded RNA virus of about thirty thousand bases in length and is easily transmitted in humans, which occurs via respiratory droplets that are produced through coughing, talking and sneezing [7]. Essentially, the SARS-CoV-2 virus has a polybasic cleavage site, a feature that is characteristic of easy transmissibility and the pathogenicity of other strains [8]. At present, the complete genome of the SARS-CoV-2 virus has been identified, and this has significantly contributed to the construction of a phylogenetic tree of the virus as well as its diagnosis and treatment.

### Hepatitis C Virus (HCV)

Hepatitis C Virus (HCV) is a small, enveloped RNA virus, and is the causative agent for the hepatitis C disease [9]. It is also known to be associated with the development of some forms of cancers such as head and neck cancer, and non-Hodgkin’s lymphoma [10]. HCV belongs to the family of Flaviviridae viruses, which are a closely related human virus class, including hepatitis G virus, dengue virus, and yellow fever virus. Structurally speaking, HCV comprises a lipid membrane and two viral envelopes, which are essential in the attachment of the virus to various surfaces [9]. The icosahedral core is located within the envelope in which the RNA material is found.

The envelope of the virus contains E1 and E2 glycoproteins, both of which play a crucial role in the interaction of the virus with the immune system [11]. Once inside the human body, the replication of HCV involves several steps – mainly in the liver cells – in a process that can produce trillions of copies of the virion in one day, even in the chronic phase of infection [12]. With increased replication rate, the virus can attack and overpower the immune system, which results in declining capabilities of an individual to respond to secondary infections.

HCV has prominent clinical importance, as it causes a disease that affects a significant number of people across the world. Based on the genetics of the virus, there are six genotypes with implications in terms of the treatment of the infection. HCV treatment often follows an interferon (INF) alpha-based treatment strategy. Of these genotypes, genotypes 1 and 4 have been shown to be less responsive to interferon-based therapy [13]. In addition, unlike with most other viral infections, where prior exposure to the virus confers immunity, exposure of an individual to a given genotype of HCV does not confer immunity toward other genotypes.

Generally speaking, HCV is transmitted via the blood and other bodily fluids. As such, the groups at high risk of contracting this disease include hemodialysis patients, blood product recipients, and intravenous drug users. Thus, hospitals are a significant setting where infections with HCV are most likely to occur. However, in different parts of the world, certain cultural factors, such as genital mutilation, acupuncture, and circumcision, could result in incidents where this condition has a higher likelihood of being transmitted.

In most cases, people infected with HCV appear to not show symptoms. Nevertheless, there is a proportion of individuals who do develop severe symptoms upon infection with this virus. In particular, the symptoms of the disease commonly include abdominal pain, loss of appetite, and diarrhea [14]. The patient may also experience jaundice. Essentially, using these symptoms alongside blood tests, healthcare professionals are often able to successfully diagnose the disease.

Even after infection, some individuals do not require treatment, as their immune system can fight and overpower the viral infection. In individuals whose immune system cannot clear the infection without outside intervention, it is necessary for different treatment options, such as siRNA treatments targeted against the viral RNA or interferon-based treatments, to be available to them. It is also important to note that, as with other COVID-19 patients with preexisting medical conditions, it is critical for individuals who have liver problems to be able to access all possible treatment options. In terms of potential treatment options, several medications are available for the treatment of HCV infection, which may also be effective for COVID-19 treatment. Antiviral medications and interferon-based therapies are the commonly-used approaches in the treatment of HCV [15]; however, genotype variations in HCV prevent the application of all these medications that have been effective against all HCV infections. As such, the healthcare professional needs to understand the patients’ hepatitis C genotype and prescribe drugs that would be effective toward the infection. This same reason may also prevent the application of HCV treatments in SARS-CoV-2 infections, should there be genotype variations.

### Similarities between HCV and SARS-CoV-2

SARS–CoV-2 belongs to the *Coronaviridae* family of viruses and HCV belongs to the *Flaviviridae* family. On the surface, SARS-CoV-2 and HCV are two different viruses, but genetically, both are positive single-strain RNA viruses (+ssRNA). Immunologically they both have similar characteristics concerning host immune responses, which might offer some insight into the treatment of COVID-19. Although not much is currently known about SARS-CoV-2, SARS-CoV has been widely studied, and an extrapolation from this might help direct possible treatment for COVID-19.

It has been reported that SAR-CoV replication is inhibited by INF-β [16]. In addition, other types of INF – namely, INF-α and INF-γ – have also been used as anti-SARS-CoV, with positive outcomes reported for *in vitro* studies [16]. Alfacon-1 corticosteroid used in the treatment of resistant-HCV was found to reduce disease-associated oxygen saturation impairment, lower the level of creatine kinase (a marker of kidney damage), and cause rapid resolution of radiographic lung abnormalities [17]. In a study by Zhao et al. [18], it was shown that SARS patients that were randomly receiving INF-α exhibited the shortest admission length, and a relatively short time for improvement of dyspnea and the clearing of chest X‐ray films when compared to those under different treatment regimens. Cinatl et al. [16] tested a series of anti-HCV drugs including 6‐azauridine, ribavirin, and glycyrrhizin as potential anti-SARS-CoV drugs, and found glycyrrhizin to be the most potent of all. Taken together, it seems that HCV and SARS-CoVs, and possibly SARS-CoV2s may have similar pathogenesis within the context of immune response.

It is known that the Th2 response is the predominant form of immune response in patients with HCV infection, and this immune response has been suspected to drive carcinogenesis in these patients [19]. Th1 and Th2 response has also be documented in patients with HCV infection [20]. Previous studies have demonstrated that during the early stage of SARS-CoV infection – usually up to 7 days post-symptoms – Th2 predominant cytokine profile characterized with increased secretion of TGFβ and IL‐13 is apparent. The Th1 predominant cytokine profile of IL‐2 and IL‐18 is apparent in the late stage – more than two weeks following symptoms [21]. This suggests that during early SARS-CoV infection, the immune response of the host may favor viral replication, which at a later stage may induce immune-mediated damage similar to the pathomechanism of SAR-CoV-2-induced airway and lung damage [22].

### Ion channel implications in viral infections

There are several factors that limit viruses’ ability to replicate, and these rely entirely on proteins of the host cells for successful propagation. In order to attack or infect, viruses have to bind to the receptors that are located on the surfaces of host cells [23]. Through this interaction, the viruses are able to enter the cell and then use the cell’s machinery to replicate and multiply. One of the facilitators of cellular entry of viruses is ion channels, which are utilized during the initial stages of infection. Ion channels are cellular proteins that facilitate the movement of ions across the cell membrane into and out of the cells [24]. This movement of ions across the channels changes electrical signals across the cell membrane and, as a result, rapid changes occur, causing changes in the intensity of intracellular messengers that regulate various cellular roles.

**Figure 1.**
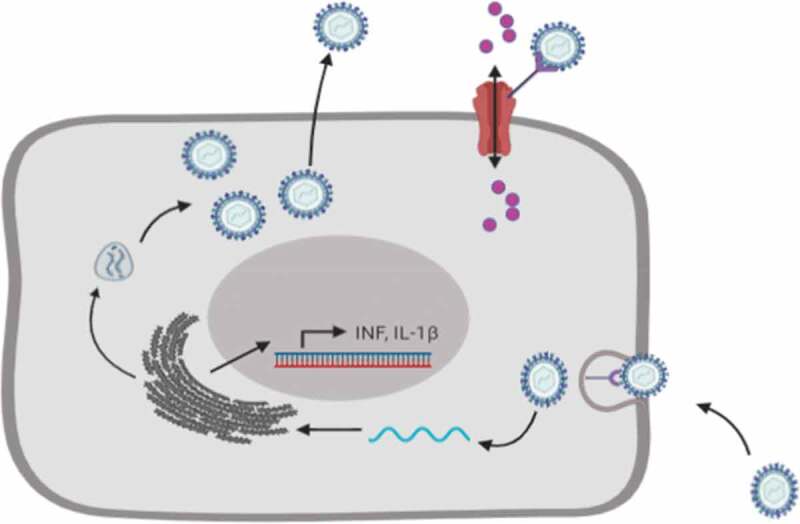
The utilization of ion channels by a virus for survival and replication. Viruses enter host cells via receptor-mediated endocytosis, after which they release their genetic material to facilitate protein synthesis using the host genetic and protein synthesis machinery in the process causing secretion of inflammatory cytokines. Viruses often interact with ion channels to facilitate entry of ions, such as Ca^2+^, to create a suitable environment for replication and survival

Usually, the virus exploits a vulnerability within a cell’s environment for survival and replication. In most cases, the virus encodes (produces) ion channels of their own, which are known as viroporins [25]. These ion channels are essential for the regulation of homeostasis during viral infection (see Figure 1 below). Nevertheless, a good number of viruses do not have viroporins, but these have developed a mechanism through which they can manipulate the ionic environment of host cells, and thus manage to survive and multiply. Essentially, different viral proteins have been found to critically affect ion channel function [26–29]. However, there are still unanswered questions as to how all these interactions connect with the various viral processes, and how this could be applied in the management of viral conditions.

Primarily based on their small size, as well as the hydrophobic nature of SARS-CoV E proteins, they are candidate members of a family of viral proteins forming ion channels [30]. Specifically, the influenza virus protein M2, the 6 K adenovirus protein, HIV-1 proteins Vpu and Vpr, and the HCV protein p7 [31] are also assumed to function as ion channels. This similarity between the SARS-CoV E proteins and the HCV p7 protein may indicate similar functioning and therapeutic targeting. The α-helical structure of the SARS-CoV E has also been determined to be similar to that of HCV p7 [32]. Based on this, both SARS-CoV E and HCV p7 proteins may modify the ionic content of the host-cell in a similar fashion, in a process that determines the success of these viruses in attacking their hosts [33].

Additionally, ion channels trigger virus production using various mechanisms, and the use of ion channel conductivity seems relevant in various cases. In addition, the disruption of ion homeostasis, especially calcium levels, has effects that enhance the rate of viral replication [34]. The key stages in the life cycle of a virus include uncoating and maturation, the latter of which is controlled by certain ion processes in most viral species. Importantly, apart from enhancing the rate of viral replication, viroporins also enhance the pathogenicity of viruses [29]. In the recent past, the pathogenicity of viruses has been connected to ion conductivity or other roles, such as encoding of ion channels or using viral proteins as ion channels, as exemplified by the SARS-CoV accessory proteins 3a and 8a [35].

Understanding ion channels could thus have a significant impact on our knowledge of viral pathogenicity. It is also possible to use this knowledge to design and develop new therapies against viruses [36]. In various cases, the developers of antiviral therapies aim to incorporate mechanisms within drugs that target ion channels. The envelope protein that SARS-CoV-2 possesses seems to comprise a transmembrane protein, and this is likely involved in the ion channel properties of the virus. Though this does not seem to influence the replication rate of the virus, it is probably involved in the pathogenesis of this virus.

### Channelopathy in HCV and COVID-19

Ion channels are pore-forming protein molecules that facilitate the passage of ions across membranes. The functions of an ion channel are influenced by their opening probability and cellular distribution [36]. Evaluation of ion channel activities during viral infection is important because virion entry, egress and sustenance of a favorable cellular environment for virus survival, in part, depends on the manipulation of the activity of ion channels by the virus [36]. Based on this, ion channels utilized by viruses have a crucial function in the regulation of virus replication. One of the ways that ion channels maintain a suitable environment for the viruses is through the mediation of the electrochemical balances in subcellular compartments in host cells, making these ion channels promising targets of anti-viral drugs [37].

Viroporins, which are ion channels used by viruses, are a small class of proteins, usually between 60 and 120 amino acids long. The main viroporins of HCV and SARS-CoV have been documented to be the p7 and the E proteins, respectively, which have been found to be structurally similar [32]. The operational significance of ion channels is underscored by the recognition that dysfunctional epithelial ion transport systems are linked to various human pathologies/diseases such as CF, COPD, pulmonary edema and chronic bronchitis [38]. These human pathologies occur because impairment of epithelial ion transport alters the contents or fluid composition of the ion channels.

Chloride (Cl^−^) channels are essential in various physiological processes such as mucus secretion, neuronal excitation, acidification of intracellular organelles, muscle contraction, transepithelial fluid transportation, and cell‐volume regulation [39]. Impairment of Cl^−^ channels is connected to numerous diseases, including myotonia, osteoporosis, and epilepsy. The most well studied disease associated with impairment of Cl^−^ channels is CF. However, Cl^−^ transport is also known to be crucial in the maintenance of HCV infection. A previous study demonstrated that HCV increases the rate of hepatocellular transport of Cl^−^, which can be inhibited by Cl^−^ channel blockers to maintain an aqueous environment for the virus [40]. Despite the significance of ion channels in virus processes, it has been found that the inhibition of Ca^2+^, Na^+,^ and K^+^ has no influence on the replication of the HCV genome [40]. Hence, Cl^−^ channels are specifically imperative for HCV’s life cycle. In a similar manner, synthetic SARS-CoV E protein has been demonstrated to also transport Na^+^, K^+^, and Cl^−^, though with low selectivity [31]. The low selectivity for specific ion transport by the SARS-CoV E protein is also shared by the HCV p7 protein [41]. It should be noted here that poor *in vitro* selectivity of both HCV p7 and SARS-CoV E proteins might be a result of incomplete specialization of these viroporins for ion transport.

### Potential targets for SARS-CoV-2 diagnosis

The levels of IFNL3 – a subset of the IFN-λ class of cytokines – in the blood can be a potential diagnostic marker for SARS-CoV-2. INFL3 is known to be a hallmark of the clearance of HCV infection and a predictor for the risk of developing fibrosis of the liver due to different hepatic diseases such chronic liver fibrosis and CF [42]. At the onset, IFNL3 possesses anti-tumorigenic and immune-modulatory effects and is primarily triggered by viral infections [43]. In addition, IFN-λ has critical immunomodulatory and anti-bacterial/viral roles across the respiratory tract. Importantly, the relationship between IFNL3 polymorphisms and circulating IFNL3 levels is dependent on the type of disease [43]. For instance, the levels of IFNL3 in serum have been found to be high in subjects with pulmonary fibrosis, resulting in the conclusion that this may be linked to both liver fibrosis and pulmonary fibrosis [42].

The level of Cl^−^ in the blood is another potential diagnosis of SARS-CoV-2. Oh et al. [44] note that Cl^−^ is the major plasma anion and interstitial fluid, representing about one-third of tonicity in plasma. Cl^−^ channels are indispensable in virus processes, including genome replication and throughout their life cycle [40]. Virus processes are thus likely to influence Cl^−^ levels in the blood. Potential diagnosis of SARS-CoV-2 can hence focus on detecting a decline in chlorine levels. In particular, the diagnosis could target hypochloremia, an imbalance of electrolytes caused by low chlorine in the blood, mainly due to Cl^−^ wasting [45]. It has also been found that hypochloremia might be a sign of illness severity [44]. Such low levels of Cl^−^ in the body might impair the functioning of epithelial Cl^−^ and sodium channel in the pulmonary system. For example, individuals with CF have a missing Cl^−^ conductance [38]. Consequently, the impairment of these channels may impact the environment in, and viral role of, the lungs. Therefore, the level of Cl^−^ can be used as an indicator of SARS-CoV-2 (COVID19 severity).

### Possible therapies targeting COVID19

SARS-CoV-2 can be targeted with a combination of two therapies: one aimed at the reduction of viral load and treatment of damages in the pulmonary system; another at the reduction of induced inflammation, both of which have been successful in treating HCV infection.

#### Ribavirin and pegylated interferon (peg-IFN)

A potential therapy is to target the virus through a combination of peg-IFN and ribavirin. Importantly, ribavirin is effective in inhibiting viral RNA-dependent RNA polymerase and can be a viable treatment for SARS-CoV-2 because of its activity against various nCoVs [46]. Specifically, ribavirin has anti-viral activity that reduces the infectivity of HCV in dose-dependent patterns [47]. The effectiveness of ribavirin against the virus can be enhanced by combining it with peg-IFN. This combination enhances the sustained virological response (SVR) rate by about 25–30% [48]. An important element in the treatment of SARS-CoV-2 is relapse prevention. It has been found that ribavirin is effective in preventing relapse among HCV patients who exhibit a response to peg-IFN antiviral effect [47]. Ribavirin prevents relapse since it reduces virus RNA gradient of transcription, which is a measure viral replication in qPCR assay. However, trials have shown that a combination of ribavirin and peg-IFN might increase the cases of adverse consequences, including chelation therapy and blood requirement [47]. Despite the suggestion that ribavirin might be a potential therapy for SARS-CoV-2, there is inconclusive data regarding its effectiveness in treating other nCoVs [46]. A combination of ribavirin and Peg-IFN, however, suggests the promise of high efficacy for combatting the virus.

#### Therapies targeted at the p7 ion channel

p7, similar to SARS-CoV-2 E protein, is an HCV protein that plays a role in viral assembly and release of mature virions. In addition to this, the p7 protein is conserved across all genotypes of HCV, making it a potentially effective therapeutic target for HCV. One of the drugs used in the treatment of viral diseases is amantadine hydrochloride, which works to inhibit viral DNA release into host cells through its interaction with the membrane M2 protein, as with the influenza virus, for instance [49]. While interferon therapy alone and with the addition to ribavirin has failed in specific HCV patients [21], amantadine inhibition of p7 ion channel activities *in vitro* and in artificial membranes has been shown to inhibit HCV infection [21]. A recent study, however, discovered that amantadine and its new analogue, rimantadine, are too small to fit the binding pocket of p7 and may not be as effective *in vivo* [50]. Developing a larger analogue of these inhibitors may thus help circumvent this potential setback and prove useful for SARS-CoV-2 treatment.

Amiloride is a guanidium compound that functions to block the Na^+^ channel in epithelial tissues to facilitate Na reabsorption in the kidney in a manner that depletes Na but not K within the body. Amiloride has been shown previously to inhibit release of viral particles in the Vpu protein of HIV-1 via inhibition of the ion channel activity that results from the virus budding [51]. Vpu has also been shown to be similar to p7 of HCV, which has prompted the investigation of amiloride activity in terms of its ability to inhibit p7. A molecular dynamic modeling study has shown that a derivative of amiloride BITT225 has strong hydrophobic interaction with p7, which may translate to *in vivo* inhibition of p7 [52]. However, this remains to be tested in human studies.

#### Other potential treatments and prevention

Increasing the concentration of NaCl in the body can have a positive impact on the management of SARS-CoV-2. Ramalingam et al. [53] assert that a high concentration of NaCl in the body enhances antiviral activity. Besides this, intracellular Cl^−^ is needed for enhanced antiviral activity. The use of selective a Cl^−^ channel blocker may also be a feasible approach to managing SARS-CoV-2. Studies indicate that HCV augments intracellular hepatic Cl^−^ influx and that selective Cl^−^ channel blockers can inhibit the influx [40]. In particular, selective inhibitors that are sensitive to the volume of Cl^−^ can help target Cl^−^ channels. Another practical therapy for SARS-CoV-2 is to target Na^+^ in the body. Schoeman and Fielding [54] found that SARS-CoV E protein exhibits more selectivity, particularly for Na^+^ compared to K^+^ ions. As a result, it is evident that the virus is sensitive to cellular environment changes, which is a vital property to understand in order to develop effective therapies based on *in vivo* and *in vitro* regulation of ionic conditions.

Therapeutic options containing hydrogen peroxide (H_2_O_2_) may also be used to treat SARS-CoV. H_2_O_2_ has been suggested because it produces various antiproliferative responses, such as mitochondrial and nuclear DNA lesions, promotes cell adhesion molecule expression, and raises p53 activity, leading to cell death [55]. It also impacts membrane protein and minimizes the invasion and migration of cells [55]. The first option is the therapeutic application of phagocytes against SARS-CoV-2. Notably, phagocytes damage ingested microbes by generating hypochlorous acid (HOCl) from Cl- ions and H_2_O_2_ in phagolysosomes, utilizing myeloperoxidase [53]. HOCl contains antiviral/antibacterial properties that aid the ingestion of microbes. The second option is the combination of 5% H_2_O_2_ and 25 μM of 1,2-bis(o-aminophenoxy) ethane-N,N,N′,N′-tetraacetic acid- acetoxymethyl ester (BAPTA-AM) [56]. This mixture can be gargled by those exposed to SARS-CoV-2 to manage sore throat during the early phases of infection. BAPTA-AM is proposed because it is an ion chelator with high specificity for Ca^2+^ [56]. The third option is to combine 5% H_2_O_2_ with 5 μM BAPTA-AM, a combination that can be administered with a nebulizer or powder inhaler. The 5 μM BAPTA-AM is an ion chelator that targets calcium ions [57]. Perhaps this approach can be helpful in the early stages of coronavirus infection.

## Conclusion

In conclusion, COVID-19 pandemic is an immense global public health crisis that threatens the core of humanity and the contemporary way of life. The severity of the viral disease and reports of relapse among recovered patients presents the need for far-reaching therapy. Importantly, the most effective treatments should target viral load reduction, symptom management, and the potential elimination of the virus from bodily cells. In this regard, this review suggests a range of potential treatments, such as Cl^−^ channel inhibitors, control of NaCl concentration, and leveraging specificity for Ca^+^ in association with H_2_O_2_, considering their ability to trigger cell death. Essentially, there is currently no effective treatment of the virus, and the suggestions presented in this paper represent promising possibilities for treating and preventing COVID-19.

## Data Availability

Data sharing is not applicable to this article as no new data were created or analyzed in this study.
